# Timing of Water Deficit Limits Maize Kernel Setting in Association With Changes in the Source-Flow-Sink Relationship

**DOI:** 10.3389/fpls.2018.01326

**Published:** 2018-10-22

**Authors:** Yebei Li, Hongbin Tao, Bingchao Zhang, Shoubing Huang, Pu Wang

**Affiliations:** College of Agronomy and Biotechnology, China Agricultural University, Beijing, China

**Keywords:** corn, water deficit, vascular bundle, yield, leaf area

## Abstract

The kernel setting of maize varies greatly because of the timing and intensity of water deficits. This variation can limit leaf productivity (source), the translocation of assimilated sugars (flow), and yield formation (sink). To explain the decline in kernel setting of maize under water deficits from the perspective of source-flow-sink, a 3-year experiment was conducted under a rain shelter. Five water regimes were studied. One regime included well-irrigated (CK) treatment. Four regimes involved water deficits: irrigation was withheld during the 6- to 8-leaf stage (V_6−8_), the 9- to 12-leaf stage (V_9−12_), the 13-leaf stage to tasseling stage (V_13−T_), and the silking stage to blister stage (R_1−2_). Water deficit effects on kernel setting began when the water deficit occurred at V_9_ and became more significant with time. Kernel weight was reduced by 12 and 11% when there were water deficits during V_9−12_ and V_13−T_, respectively. This was the result of reduced leaf area (limited source) and an altered vascular bundle in the ear peduncles (limited assimilate flow). The reduced vascular bundle number, rather than the ear peduncle cross-sectional area, significantly affected the final kernel weight when exposed to a water deficit prior to the silking stage. The water deficits prior to and close to the flowering stage significantly reduced ear kernel number; that is, 14 and 19% less during V_13−T_ and R_1−2_, respectively, compared with the kernel number during the CK treatment. This reflects a smaller sink under water deficit conditions. Additionally, ovary size was reduced the most in the V_13−T_ water deficit compared with other treatments. After rewatering, the water deficit before or during flowering stage continued to have residual effects on grain-filling in the late growth period. The grain-filling rate decreased under the V_9−12_ water deficit; the grain-filling duration shortened under the R_1−2_ water deficit; and both negative effects occurred under the V_13−T_ water deficit. This study clearly indicated that (1) the water deficit during the vegetative organ rapid growth period both limited leaf source development and assimilate flow and slowed down kernel development, and (2) the water deficit just before and during flowering reduced kernel sink. Deficits at both times could retard grain-filling and reduce maize yield. The results of the present study might guide irrigation practices in irrigated maize or inform the management of sowing time in rainfed maize, to desynchronize the water deficit and the plant’s reactions to such deficits at different stages.

## Introduction

Maize is the most important cereal crop in the world, and maize production faces great challenges in increasing or maintaining yields, based on severe abiotic stresses that are brought about by climate change (Lobell et al., 2011; Lesk et al., 2016). The most severe abiotic stress restricting maize yields is the presence of water deficits; these deficits account for approximately 50% of total maize yield (tons) loss due to abiotic stresses in China (China Statistical Yearbook, 2016).

Maize grain yield is sensitive to water deficits (Schussler and Westgate, 1991; Setter et al., 2001). Kernel number and kernel weight are the most impacted components (Borrás and Gambín, 2010). The kernel number is more flexible and is more closely correlated with grain yield than the kernel weight, especially under abiotic stress (Andrade et al., 2002). Kernel numbers can be reduced by up to 100% because of water deficits, depending on the time and intensity of the deficits (Schussler and Westgate, 1991; NeSmith and Ritchie, 1992a; Cakir, 2004). The loss of kernel numbers under a water deficit can be attributed to incompletely developed florets (Rossini et al., 2016), inhibited silk emergence (Fuad-Hassan et al., 2008; Oury et al., 2016), and zygotic abortion (Zinselmeier et al., 1999; McLaughlin and Boyer, 2004a). In contrast, kernel weight is more stable under a water deficit, and any declines usually occur during the late growth period because of the reduced leaf area (NeSmith and Ritchie, 1992b; Mansouri-Far et al., 2010). The reduction of kernel weight under a water deficit is related to impaired assimilation supply (Schussler and Westgate, 1995; Setter et al., 2001), disturbed sugar and hormonal metabolism (Ober et al., 1991; Zinselmeier et al., 1999), and decreased cell division and starch accumulation in the endorsperm (Setter and Flannigan, 2001; Yu and Setter, 2003). In other crops, kernel weight also relates to the development of parental organs prior to flowering (Yang et al., 2009; Hasan et al., 2011; Castillo et al., 2017); however, there is little relevant research on maize.

Kernel setting is associated with a source-sink relationship (Borrás and Otegui, 2001; Borrás et al., 2003b); this relationship is an important determinant of maize yield (Borrás and Gambín, 2010; Yu et al., 2015). Source activity and sink capacity can be affected by water deficits by reducing leaf area, accelerating leaf senescence (Prochazkova et al., 2001), and decreasing leaf photosynthetic rate (*Pn*) and its associated metabolism (Setter et al., 2001). The limited sucrose levels under water stress decrease the number of endosperm cells and starch granules, thereby, reducing sink capacity (Ober et al., 1991; Setter and Flannigan, 2001). The source and sink are not affected independently by water deficits; rather, they restrict each other. Carbohydrate shortages result in kernel abortion and fewer kernels. This can impair sink strength (Zinselmeier et al., 1999; McLaughlin and Boyer, 2004b). Consequently, the decreased kernel capacity results in sugar accumulation in the leaf and the stem, especially post-silking, which downregulates photosynthesis in the leaf (Rossi et al., 2015). The altered source-sink relationship reduces plant biomass accumulation and reduces the translocation of assimilated sugars to the kernels (Jurgens et al., 1978). When considering source-sink relationships under water deficits, the flow of assimilate from source to sink is significantly reduced under water deficits. However, more relevant evidence is needed to demonstrate these outcomes.

Therefore, this study analyzed the causal factors that reduce maize yield under water deficits in terms of source, assimilate transport, and sink, both separately and together as a system. To achieve this objective, we measured (1) grain yield and yield components; (2) the leaf area in different canopy layers and ear leaf photosynthesis at mid grain-filling; (3) vascular bundle size and number in the ear peduncle; and (4) ovary development and grain-filling dynamics. The study was conducted over 3 years under different water deficit treatments.

## Materials and Methods

### Experimental Site and Agronomic Management

The experiments were conducted in a rain shelter at the Shangzhuang Experimental Station at the China Agricultural University, Beijing, China (40° 08′N, 116° 10′E) during 2014–2016. The rain shelter contained 18 big ponds (2 m long, 4 m wide, and 1.8 m deep) and 18 small ponds (2 m long, 2 m wide, and 1.8 m deep). Each pond was cemented on the four sides and the bottom, and each pond was filled with calcareous fluro-aquic soil. The soil contained 9240 mg kg^−1^ organic matter, 519 mg kg^−1^ total nitrogen (TN), 68.3 mg kg^−1^ extractable potassium (K_2_O), and 16.6 mg kg^−1^ extractable phosphorous (P) at soil depths of 0–40 cm. The field capacity was 0.32 g g^−1^ at a soil depth of 0–20 cm and 0.25 g g^−1^ at a soil depth of 20–40 cm. The soil bulk density was 1.38 g cm^−3^ at a soil depth of 0–20 cm and 1.59 g cm^−3^ at a soil depth of 20–40 cm.

Prior to sowing, 60 kg N ha^−1^, 90 kg phosphorus pentoxide (P_2_O_5_) ha^−1^, and 150 kg K_2_O ha^−1^ were applied evenly to the soil surface. The fertilizer together with soil was then plowed under with a shovel, and an additional 120 kg N ha^−1^ was applied by furrowing at the 12-leaf stage (V_12_). The most popular maize hybrid *Zhengdan* 958 in China was used. Seeds were planted on June 10th in all 3 years and were harvested after the seeds reached physiological maturity. Three to four seeds per hole were sowed. The seedlings were thinned to 7.5 plants m^−2^ at the 3-leaf stage (V_3_) with a row spacing of 60 cm and plant spacing of 20 cm. Weeds, pests, and diseases were well controlled during the experimental seasons.

### Experimental Design

The experiment was arranged in a completely randomized block design with three replications in 2014 and four replications in 2015 and 2016. Five treatments were conducted: (1) well-irrigated (CK) treatment with irrigation at the 6-leaf (V_6_), 9-leaf (V_9_), 13-leaf (V_13_), silking (R_1_), and blister (R_2_) stages; (2) introducing a water deficit during the 6–8-leaf stage (V_6−8_) by skipping irrigation once at V_6_; (3) introducing a water deficit during the 9–12-leaf stage (V_9−12_) by skipping irrigation once at V_9_; (4) introducing a water deficit during the 13-leaf stage to tasseling stage (V_13−T_) by skipping irrigation once at the V_13_ stage; and (5) introducing a water deficit during the silking stage to blister stage (R_1−2_) by skipping irrigation once at silking. Full irrigation was applied approximately 10 days before sowing each year. During the maize growth season, for the CK treatment, the soil water content was maintained above 50% of field capacity at a soil depth of 0–60 cm (Andrade et al., 2002). For the four water deficit treatments, the water content dropped below 50% of field capacity during the water deficit period associated with the different treatments. The relative soil water content is shown in Supplementary Figure S1.

### Sampling and Measurements

#### Relative Soil Water Content

Soil samples were collected using an auger at three different soil layer depths (0–20, 20–40, and 40–60 cm) to determine the water content at 1 or 2 days before V_6_, V_9_, V_13_, R_1_, and R_2_ from 8 big ponds and 8 small bigs (2 replication per pond). The soil samples were oven dried at 105°C for 24 h. Soil water content (SWC) was calculated using the following formula:

SWC = [(Fresh ​weight−Dry weight)/(Dry weight)] × 100%

The relative soil water content (RSWC) was calculated using the following formula:

RSWC = [(Soil Water Content)/(Field Capacity)] ×100%

The amount of irrigation (IW) was modified according to the method described by Tari (2016). The total irrigation amount was calculated by adding the IW for the three different soil layers (0–20, 20–40, and 40–60 cm). The IW at each layer was calculated using the following formula:

IW = 1000 BDDA (80% FC − SWC)

In this expression, IW is the amount of irrigation at each layer (m^3^); BD is the soil bulk density (kg m^−3^); D is the depth of each soil layer (0.2 m); A is the area of each plot (m^2^); FC is field capacity; and SWC is the gravimetric soil water content.

#### Leaf Area and Photosynthetic Rate

Three plants in 2014 and 2015, six plant in 2016 were randomly selected in each treatment at the tasseling stage to measure the length and width of each leaf. The leaf area was calculated as the leaf length × leaf width × 0.75 (Pearce et al., 1975). We recorded the leaf area at the “ear layer” (including ear leaf, one leaf above it, and one leaf below it), at the “above ear layer” (all leaves above ear layer), and at the “ear and above layer” (including leaves at both ear and above ear layers) (Supplementary Figure S2). The leaf area for each of these three layers was calculated by adding its included leaf areas (Subedi and Ma, 2005; Ning et al., 2017). The reduction of leaf area was expressed as the percentage of leaf area reduction at a specific layer for the plants exposed to the water deficit treatments compared with the plants exposed to the CK treatment.

Another three plants were randomly selected in each plot at the mid grain-filling stage [85 days after sowing (DAS)] in 2016. The net *Pn* of the ear leaf of each plant was measured with LI-6400 XT (Li-Cor Inc., Lincoln, NE, United States) at 10:00–12:00 am. Prior to measuring the leaf *Pn*, 400 μmol mol^−1^ CO_2_ and 500 μmol s^−1^ flow rate were set. The light flux density and temperature in the leaf chamber were consistent with ambient conditions.

#### Vascular Bundle and Ear Peduncle

Ear peduncles next to the base of the ears were sampled twice, one was just before tasseling (49 DAS), another is 10 days after silking (65 DAS) in 2016. The peduncles collected at 49 DAS were immediately fixed after sampling with a mixture of 10% formaldehyde, 50% ethanol, and 5% acetic acid over 24 h in a refrigerator at −4°C. The samples were then dehydrated solutions with different concentrations of ethanol: 75% ethanol for 4 h, 85% ethanol for 2 h, 90% ethanol for 2 h, 95% ethanol for 1 h, and twice in 100% ethanol for 0.5 h. The dehydrated peduncles were infiltrated with mixtures of xylene and alcohol five times (ethanol:xylene (v/v) = 2:1 for 10 min once, ethanol:xylene = 1:1 for 10 min once, ethanol:xylene = 1:2 for 10 min once, and then 100% xylene for 10 min twice). Afterward, the ear peduncles were embedded in paraffin wax and were cut into 4 μm sections using a Leica RM 2016 microtome (Leica Shanghai Instrument Co., Ltd., Shanghai, China). Finally, the peduncle sections were de-waxed and stained with 0.5% safranin and 0.5% fast green according the method described by Liu et al. (2006). Micrographs were taken using a Nikon Elipse Ci (Nikon Instruments Inc., China) and then analyzed using a CaseViewer (3DHISTECH Ltd. The Digital Pathology Company).

Cross sections of three ear peduncles were collected from big ponds (3 replicates) at 65 DAS. Razor blades were used to cut freehand slices (Xu et al., 2017), which were then stained with safranin (0.5%, w/v). Micrographs of the cross sections were taken with an Olympus SZ61 stereomicroscope (Olympus Imaging China Co., Ltd., Beijing, China). Vascular bundle size and number and ear peduncle area were measured using an Image-Pro Plus 6.0 (Media Cybernetics Inc., 2006).

#### Ovary Size

Fresh ovaries at the 5th kernel ring of the ear were manually cut longitudinally through the middle of the embryo (Leroux et al., 2014). The samples were then stained with 0.05% toluidine blue O for 15 min. The samples were then rinsed with distilled water for 1 min. Micrographs were taken with an Olympus SZ61 stereomicroscope.

#### Grain-Filling and Its Characteristics

The ears of 18 plants were randomly selected for each treatment and then bagged before silking occurred in 2015. Artificial pollination was conducted 4 days after silking according to the method described by Cárcova and Otegui (2001). Three representative ears in each treatment were harvested to measure kernel weight at 5, 15, 25, 35, 45, 60 days after pollination.

Kernels were classified as either inferior or superior based on their position in the ear. After sampling, the ears were cut into two parts at a point that was located at two-thirds of the distance between the bottom of the ear and the top. The kernels from the top one-third of the ear were defined as inferior kernels, while the rest were defined as superior kernels. Four full rows of kernels from the top one-third of the ear and two full rows of kernels from the bottom two-thirds of the ear were collected separately and dried to a constant weight at 70°C to determine kernel dry weight (Gao et al., 2017).

The grain-filling process was divided into three periods according to the study by Borrás and Gambín (2010): lag period, the active grain-filling period, and the maturation drying period. The three grain-filling periods were determined by calculating the second derivative of a logistic growth equation [W = a/(1 + be^−ct^)], where ‘W’ represents kernel weight (mg); ‘a’ represents the potential kernel weight; ‘b’ and ‘c’ are coefficients, which were determined by regression; and ‘t’ represents the number of days after pollination (Wang et al., 2010). The characteristic parameters of the grain-filling rate (V_lag_, V_active_, and V_drying_) and the duration (T_lag_, T_active_, and T_drying_) of each grain-filling period were calculated based on the derivative of the logistic growth equation (Raynaud, 2010; Gao et al., 2017). The starting time of the active grain-filling period was defined as *t*_1_ = [ln b – ln (2 + 3^1/2^)]/c and ending time was *t*_2_ = [ln b + ln (2 + 3^1/2^)]/c (Supplementary Figure S3). The duration of the entire grain-filling process was *t*_3_ = (ln b + 4.595)/c. Therefore, the duration of lag period (T_lag_) was T_lag_ = t_1_, active grain-filling period (T_active_) was T_active_ = *t*_2_ – *t*_1_, and maturation drying period (T_drying_) was T_drying_ = *t*_3_ – *t*_2_. The corresponding grain-filling rates of lag period (V_lag_), active grain-filling period (V_active_), and maturation drying period (V_drying_) were the daily kernel weight increase during its corresponding duration.

#### Grain Yield and Yield Components

All the ears from each plot were harvested, counted, and weighed once physiological maturity was reached. From these ears, 20 representative ears from big ponds and 15 ears from small ponds during 2014, 20 representative ears from all ponds during 2015, and the rest of the ears from all the ponds during 2016 were selected for each plot. The row number and kernel number of the two opposite rows of each ear were counted to determine the kernel number per ear. Kernels from these ears were then manually threshed and oven-dried at 70°C to a constant weight. Grain yield was calculated based on the dry kernel weight of each ear and ear number in each plot. The yield was adjusted to 14% moisture content. In addition, three replicates of 1000 kernels in each plot were counted, oven-dried, and weighed to determine the 1000 kernel weight.

### Data Analysis

Analysis of variance (ANOVA) was conducted using the SAS 9.0 statistical package (SAS Institute, 2004). The least significant difference (LSD) was used to determine significant differences among treatments at the 0.05 probability level. Figure drawing, grain-filling curve fitting, and correlation analysis were performed using SigmaPlot 12.5 (Systat Software Inc., 2013).

## Results

### Grain Yield and Yield Components

The timing of water deficit significantly affected the maize yield and yield components (Table 1 and Supplementary Table S1). The maize yield significantly decreased by 8.3% (*P* < 0.001) as a result of the V_6−8_ water deficit, compared with the result of the CK treatment. There were no significant differences in the kernel number and thousand kernel weight (TKW) when comparing the V_6−8_ water deficit treatment and the CK treatment. Both the kernel number and TKW significantly decreased as a result of the V_9_–R_1_ water deficit; this led to a significant decline in maize yield (23.8–33.7%). In addition, the lowest TKW was observed as a result of the V_9−T_ water deficit; the smallest kernel number was observed as a result of the V_13_-R_2_ water deficit.

**Table 1:** Average reduction of maize grain yield (%), kernel number per ear (%), and kernel weight (%) that resulted from water deficits during 2014–2016.

Treatment	Grain yield reduction (%)	Kernel number reduction per ear (%)	1000-kernel weight reduction (%)
CK	–	–	–
V_6−8_	8.9^∗∗∗^	5.1 ns	2.9 ns
V_9−12_	23.8^∗∗∗^	9.4^∗∗^	12.2^∗∗∗^
V_13−T_	26.7^∗∗∗^	14.0^∗∗∗^	11.1^∗∗∗^
R_1−2_	33.7^∗∗∗^	19.1^∗∗∗^	3.8^∗∗^
**ANOVA**			
Year	^∗∗∗^	^∗∗∗^	^∗∗∗^
Water deficit	^∗∗∗^	^∗∗∗^	^∗∗∗^
Year × Water deficit	ns	ns	ns

*CK, well-irrigated treatment; V_6−8_, water deficit during the 6–8-leaf stage; V_9−12_, water deficit during the 9–12-leaf stage; V_13−T_, water deficit from the 13-leaf stage to tasseling stage, R_1−2_, water deficit from the silking stage to blister stage. ^∗∗^ and ^∗∗∗^ indicate significance at P < 0.01 and P < 0.001, respectively; ns indicates no statistically significant relationship.*

### Leaf Area at Different Layers and Photosynthetic Rate at Mid Grain-Filling Stage

**FIGURE 1:**
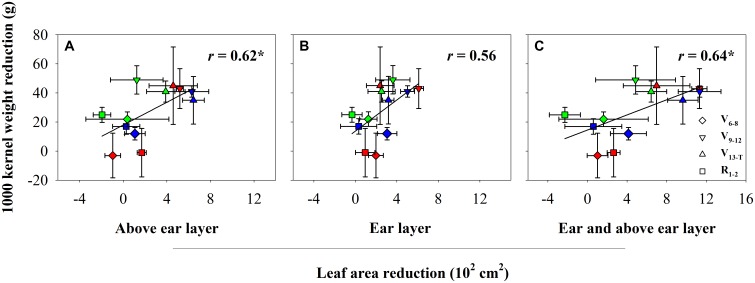
The relationship between kernel weight and leaf area reduction at above ear layer **(A)**, ear layer **(B)**, and ear and above ear layer **(C)** in 2014 (red), 2015 (blue), and 2016 (green). CK, well-irrigated treatment; V_6−8_, water deficit during the 6–8-leaf stage; V_9−12_, water deficit during the 9–12-leaf stage; V_13−T_, water deficit from the 13-leaf stage to tasseling stage, R_1−2_, water deficit from the silking stage to blister stage. ^∗^indicates significance at *P* < 0.05.

The water deficit treatments led to reductions in maize leaf area at the different layers. The reduction depended on water deficit timing (Table 2 and Supplementary Table S1). The V_6−8_ water deficit led to a significant decrease in leaf area in the ear layer, 7.9% (*P* < 0.001), compared with the leaf area observed with the CK treatment. The V_9−T_ water deficit led to a significant decrease in leaf area for both the ear and above ear layers (*P* < 0.001). However, the leaf area reduction at the ear layer was greater as a result of the V_9−12_ water deficit compared with the V_13−T_ water deficit (16.8% vs. 10.5%). In contrast, an opposite trend was observed at the above ear layer, with the V_13−T_ water deficit leading to a greater reduction in leaf area (35.4%) compared with the V_9−12_ water deficit (20.3%). No leaf area reduction was observed as a result of the R_1−2_ water deficit compared with the leaf area under the CK treatment. The TKW reduction was positively correlated with the leaf area reduction, and the correlation became significant for leaf area reduction at above ear layer as well as with the ear and above ear layers (Figure 1). However, no significant correlation was observed between leaf area reduction and kernel number reduction (data not shown).

**Table 2:** Average reduction of leaf area (%) at the ear layer, the above ear layer, and the ear and above layers around the tasseling stage that resulted from the water deficits during 2014–2016.

Treatment	Ear layer (%)	Above ear layer (%)	Ear and above layer (%)
CK	–	–	–
V_6−8_	7.9^∗∗∗^	1.2 ns	6.0 ns
V_9−12_	16.8 ^∗∗∗^	20.3^∗∗∗^	20.8^∗∗∗^
V_13−T_	10.5^∗∗∗^	35.4^∗∗∗^	22.2^∗∗∗^
R_1−2_	0.6 ns	−5.8 ns	−1.9 ns
**ANOVA**			
Year	^∗∗∗^	^∗∗∗^	^∗∗∗^
Water deficit	^∗∗∗^	^∗∗∗^	^∗∗∗^
Year × water deficit	ns	ns	ns

*CK, well-irrigated treatment; V_6−8_, water deficit during the 6–8-leaf stage; V_9−12_, water deficit during the 9–12-leaf stage; V_13−T_, water deficit from the 13-leaf stage to tasseling stage, R_1−2_, water deficit from the silking stage to blister stage. ^∗∗^ and ^∗∗∗^ indicate significance at P < 0.01 and P < 0.001, respectively; ns indicates no statistically significant relationship.*

**FIGURE 2:**
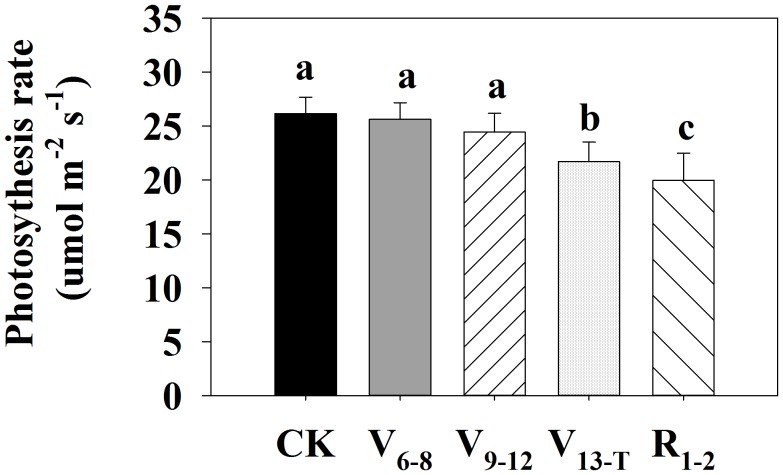
Photosynthetic rate at 85 days after sowing (mid grain-filling stage) in 2016. CK, well-irrigated treatment; V_6−8_, water deficit during the 6–8-leaf stage; V_9−12_, water deficit during the 9–12-leaf stage; V_13−T_, water deficit from the 13-leaf stage to tasseling stage, R_1−2_, water deficit from silking stage to blister stage. Values with different letters are significantly different at *P* < 0.05.

The effects of water deficits on ear leaf *Pn* at the mid grain-filling stage strongly depended on water deficit timing (Figure 2). The V_6−8_ and V_9−12_ water deficits led to similar *Pn* values compared with the CK treatment. In contrast, water deficits after V_13_ significantly reduced ear leaf *Pn* at the mid grain-filling stage, especially for the R_1−2_ water deficit.

### Vascular Bundle at Ear Peduncle

**FIGURE 3:**
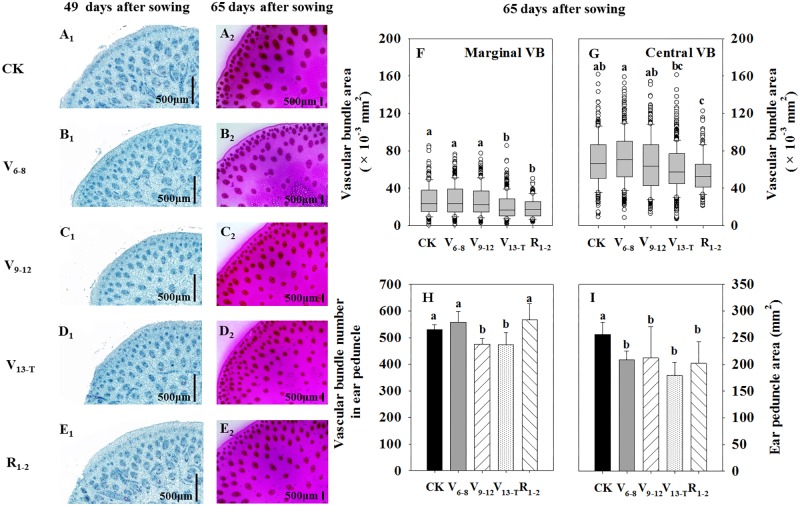
Transverse section of ear peduncle at 49 **(A_1_–E_1_)** and 65 **(A_2_–E_2_)** days after sowing (DAS); the area of the marginal vascular bundle (VB) **(F)** and central VB **(G)**; and the total vascular bundle number in ear peduncle **(H)** and ear peduncle area **(I)** at 65 DAS (12 days after silking). The marginal VB, closely arranged at the edge of the ear peduncle, was consistently undeveloped and was small in size. The central VB, scattered and distributed in the center of the ear peduncle, was consistently well-developed and had a large size. CK, well-irrigated treatment; V_6−8_, water deficit during the 6–8-leaf stage; V_9−12_, water deficit during the 9–12-leaf stage; V_13−T_, water deficit from the 13-leaf stage to tasseling stage, R_1−2_, water deficit from the silking stage to blister stage. Values with different letters are significantly different at *P* < 0.05.

The size and number of vascular bundles of ear peduncles were reduced to some extent under water deficits; the reductions were associated with water deficit timing (Figure 3). Vascular bundle development was delayed as a result of V_6−8_ (Figure 3B_1_) and V_9−12_ (Figure 3C_1_) water deficit treatments compared with the CK treatment (Figure 3A_1_) at 49 DAS; however, the vascular bundle size became similar to that observed with the CK treatment (Figures 3A_2_–C_2_,F,G) at 65 DAS. The V_13−T_ water deficit treatment postponed the vascular bundle development at 49 DAS (Figures 3A_1_,D_1_) and significantly decreased the vascular bundle size at 65 DAS. This was particularly the case for marginal vascular bundles (Figures 3A_2_,D_2_,F,G). With the R_1−2_ water deficit treatment, vascular bundle development was similar to that of the CK treatment at 49 DAS (Figures 3A_1_,E_1_); however, the vascular bundle size decreased significantly at 65 DAS (Figures 3A_2_,E_2_,F,G). The number of vascular bundles decreased significantly under the V_9_-V_T_ water deficit treatment (Figure 3H), while the ear peduncle area decreased when the water deficit occurred after V_6_ (Figure 3I).

**FIGURE 4:**
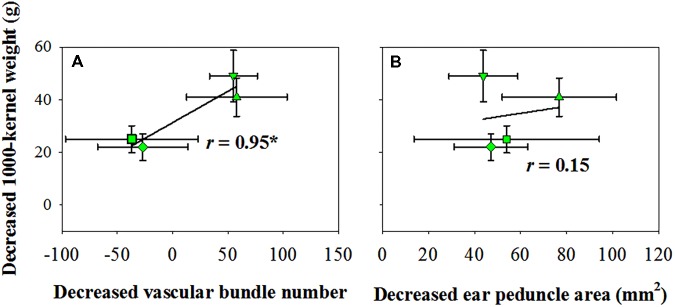
Correlation of kernel number reduction with vascular bundle number reduction **(A)** and ear peduncle area reduction **(B)** in 2016. CK, well-irrigated treatment; V_6−8_, water deficit during the 6–8-leaf stage; V_9−12_, water deficit during the 9–12-leaf stage; V_13−T_, water deficit from the 13-leaf stage to tasseling stage, R_1−2_, water deficit from the silking stage to blister stage. ^∗^indicates significance at *P* < 0.05.

The vascular bundle number reduction was positively and significantly correlated with the reduction in TKW when exposed to water deficits (Figure 4A); however, the ear peduncle area reduction had no significant effects on TKW reduction (Figure 4B).

### Ovary Development and Grain-Filling Dynamics

**FIGURE 5:**
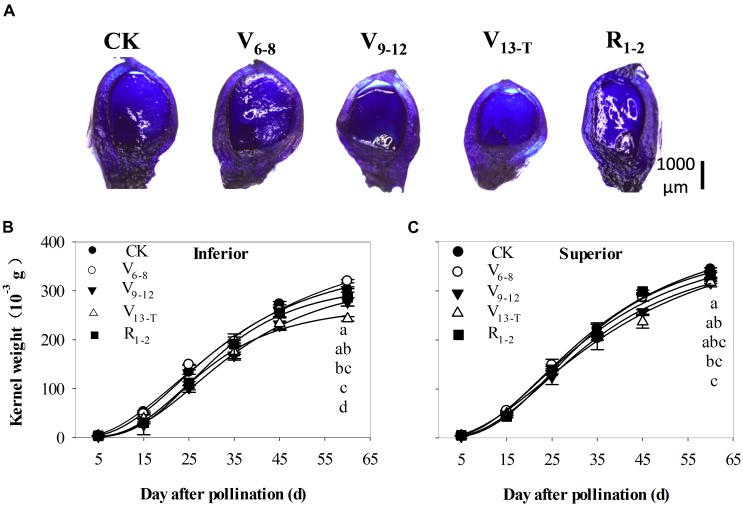
Longitudinal-sectional micrographs **(A)** of the ovary at 5th kernel ring, stained with toluidine blue at 4 days after pollination, and grain-filling dynamics of inferior **(B)** and superior **(C)** kernels in 2015. CK, well-irrigated treatment; V_6−8_, water deficit during the 6–8-leaf stage; V_9−12_, water deficit during the 9–12-leaf stage; V_13−T_, water deficit from the 13-leaf stage to tasseling stage, R_1−2_, water deficit from the silking stage to blister stage. Values with different letters are significantly different at *P* < 0.05.

The ovary size became smaller with delays in the water deficit prior to the silking stage (V_6_–V_T_). Ovary sizes recovered slightly when exposed to the water deficit after silking (R_1−2_) (Figure 5A). Ovary development was delayed as a result of the V_9−T_ water deficit compared with ovary development during the CK treatment at 4 days after pollination (Figure 5A). Accordingly, the final kernel weight was smallest for the V_9−12_ and V_13−T_ water deficit treatments (Figures 5B,C), especially for the inferior kernels. When the water deficit occurred during the early (V_6−8_) and late (R_1−2_) periods, there were no effects or slight effects on TKW.

The logistic equation fit well with the grain-filling process when exposed to the water deficit based on their high correlation values, ranging from 0.989 to 0.999 (data not shown). The timing of water deficit affected grain-filling dynamics by changing the duration of grain-filling and the grain-filling rate, especially for inferior kernels as a result of the V_13−T_ and R_1−2_ water deficit (Table 3). Furthermore, the effective filling period for the inferior kernels was shortened by 3.2–3.4 days under the V_13_–R_2_ water deficit, while the maturation drying period decreased by 4.0–4.3 days compared with the CK treatment. There was less than a one-day variation in the grain-filling duration under the V_6_–V_12_ (V_6−8_ and V_9−12_) water deficit treatment. In contrast, the grain-filling rate changed tremendously in both superior and inferior kernels. For superior kernels, the grain-filling rate during the lag period decreased more significantly as a result of the V_9−12_ water deficit (11.9%) treatment, whereas the grain-filling rate during the two late periods decreased more significantly as a result of the V_13−T_ water deficit treatment (15.0%) compared with the CK treatment. For inferior kernels, the greatest reduction in grain-filling rate during lag period was observed as a result of the V_13−T_ water deficit treatment (22.2%). The greatest reduction in the effective filling period (6.7%) and the maturation drying period (6.6%) occurred as a result of the V_9−12_ water deficit treatment.

**Table 3:** Characteristic parameters of grain-filling curve fitted by using the logistic equation, duration of lag periods (P_lag_), active grain-filling period (P_active_), maturation drying period (P_drying_), grain-filling rate during lag periods (V_lag_), active grain-filling period (V_active_), and maturation drying period (V_drying_) that resulted from the water deficit in 2015.

Treatment	Position	P_lag_ (days)	V_lag_ (mg d^−1^ kernel^−1^)	P_active_ (days)	V_active_ (mg d^−1^ kernel^−1^)	P_drying_ (days)	V_drying_ (mg d^−1^ kernel^−1^)
CK	Inferior	17.4	3.8	22.9	7.8	28.5	2.3
V_6−8_		17.0	4.0	25.4	7.4	31.6	2.2
V_9−12_		20.3	3.0	22.3	7.3	27.8	2.1
V_13−T_		17.9	2.9	19.5	7.4	24.2	2.2
R_1−2_		19.7	3.1	19.7	8.5	24.5	2.5
CK	Superior	17.5	4.2	23.8	8.4	29.6	2.5
V_6−8_		17.1	4.1	23.9	8.0	29.8	2.4
V_9−12_		18.1	3.7	23.0	8.0	28.7	2.3
V_13−T_		17.2	3.9	25.6	7.2	31.9	2.1
R_1−2_		18.8	3.8	22.2	8.9	27.6	2.6

*CK, well-irrigated treatment; V_6−8_, water deficit during the 6–8-leaf stage; V_9−12_, water deficit during the 9–12-leaf stage; V_13−T_, water deficit from the 13-leaf stage to tasseling stage, R_1−2_, water deficit from the silking stage to blister stage.*

## Discussion

The balance between source and sink during grain-filling is of great importance for kernel setting (Jones and Simmons, 1983; Borrás et al., 2002). This balance can be influenced by water deficits, especially around the pollination period (Zinselmeier et al., 1995; Setter et al., 2001). The assimilation supply increases the kernel setting by feeding sucrose during water deficits around the pollination period (Zinselmeier et al., 1999; McLaughlin and Boyer, 2004a,b). This study found that the reduced leaf area, accelerated leaf senescence, and decreased photosynthesis resulting from water deficits reduced leaf source activity, negatively affected kernel setting, and reduced maize yield (Tables 1, 2 and Figure 2). The reduction of vascular bundle size and number at the ear peduncle when exposed to water deficit treatments also limited the transportation of sucrose (Figure 3). This represented a limited potential capacity of assimilate flow.

The study also found a slower ovary development, a shortened duration of grain-filling, and a decreased grain-filling rate as a result of water deficit treatments. This resulted in a reduction in kernel weight, especially for inferior kernels (Table 3 and Figure 5). Both the decline in source activity and sucrose flow strength hindered the development of the kernels and decreased the kernel dry weight, especially for the inferior kernels (Figure 5 and Table 3). Water deficits impacted kernel size and kernel number by altering source strength (Table 2 and Figure 2), the ability of assimilate flow (Figure 3), and sink capacity (Setter and Flannigan, 2001; Borrás and Westgate, 2006). The timing of the stress governed which component was most affected (Table 1).

### Source: Leaf Area Dynamics and Photosynthetic Rate During Grain-Filling

Leaf area significantly decreased as a result of water deficits (Table 2); this decline was closely associated with water deficit timing (Cakir, 2004). In this study, the leaf area at both ear and above ear layers decreased significantly as a result of water deficits during the rapid growth period (V_9_-V_T_, Table 2). This decreased kernel weight and reduced grain yield by more than 20% (Table 1) were consistent with the findings of NeSmith and Ritchie (1992a). Previous results also indicated that water deficits occurring during the development of leaves in the ear and above ear layers could greatly reduce yields, due to the significant contribution of these leaves to grain yield (Allison and Watson, 1966; Subedi and Ma, 2005).

In addition to impacting leaf development, water deficits also accelerate leaf senescence, especially after silking (Brevedan and Egli, 2003; Sade et al., 2017). As a consequence, green leaf duration is also reduced, limiting the availability of assimilate under water deficits (Chaves et al., 2002; Borrás et al., 2003a). Boyle et al. (1991) and Zinselmeier et al. (1999) indicated that carbon starvation in maize kernel, due to limited assimilation, could result in ovary abortion, reducing the kernel number. The reduction in sink strength as a result of water deficits could result in redundant sugars in the leaf, which could trigger leaf senescence (Adams et al., 2013; Rossi et al., 2015). There was likely feedback from sink limitations on assimilation, transport, and vascular changes; however, there is a lack of relevant evidence to demonstrate this.

This study confirms that water deficits can reduce leaf *Pn*, with persistent effects. Water deficits occurring around the silking stage, especially during the post-silking stage, can reduce the *Pn* in the ear leaf at a late growth stage, even in well-watered conditions (Figure 2). This indicates that the recovery of *Pn* from water stress around the silking stage is difficult or incomplete. This outcome is consistent with the results of Grzesiak et al. (2006), probably because of the damaged enzymes of the photosynthetic system (Tezara et al., 1999; Chaves et al., 2002; Ali and Ashraf, 2011) and the accelerated leaf senescence (Escobar-Gutiérrez and Combe, 2012).

### Potential Flow of Assimilate: Vascular Bundle Size and Number in the Ear Peduncle Limits Transportation as a Result of the Water Deficit

The long-distance transportation of sucrose in maize depends on the vascular bundle system that connects leaf vein, stem, and ear peduncle (Ruan et al., 2010; Baker et al., 2016). The vascular bundle in the ear peduncle represents the end of sucrose transportation for ear growth. Larger sizes and an increased number of the vascular bundles in the ear peduncle benefit kernel setting, especially for kernel weight (He et al., 2005, 2007). This study found that the size and/or number of vascular bundles in the ear peduncle were significantly reduced as a result of the water deficit. This was particularly true when the water deficit occurred after V_9_ (Figure 3). On the one hand, the smaller size and the less number of the vascular bundles likely contributed to the reduced ability of the xylem to transport water and nutrients from the soil to the plant. This could affect the assimilation transport ability and then organ development (Tanguilig et al., 1987; Otegui et al., 1995; Hu et al., 2008). On the other hand, the limited vascular bundle system could decrease daily flow of assimilate from leaves to kernels, reducing the available sugar accumulation in the kernel (Wang, 2011; Feng, 2014). Moreover, the changes in the vascular bundles likely resulted in the incomplete recovery of leaf *Pn* and kernel setting in the late growth period of maize (Zinselmeier et al., 1999; McLaughlin and Boyer, 2004a,b). The results of the present study suggested that the water deficit had continuous effects on assimilate transportation, even after rewatering. This was due to the changes in the size and number of the flow system components responsible for assimilation in maize. In particular, the water stress occurring during the ear growth stage between the 9-leaf stage and approximately 2 weeks after pollination appears to have had larger effects on the size and number of flow system components. This was due to the important contribution of leaves to expanded yields during this growth period (Figure 3 and Tables 1, 2).

### Sink: Hampered Kernel Development and Influenced Grain-Filling

Water deficits during the silking stage greatly affected maize yield, mainly by reducing kernel number (Table 1) in association with pollen amount and activity (Uribelarrea et al., 2002; Aylor, 2004), the anthesis-silking interval (Bolaños and Edmeades, 1990, 1996), and embryo and endosperm development (Borrás and Gambín, 2010; Leroux et al., 2014). All these factors could be influenced by water deficits at the silking stage, thereby, greatly reducing the kernel number. In contrast, kernel weight was less sensitive to water deficits during R_1−2_. This might be because of the relatively high distribution of assimilate to individual kernels and the lower kernel number under severe water stress (Table 1). These results indicated that maintaining a high kernel number was the key to reducing or eliminating water deficit effects at the silking stage of maize production.

Several studies have examined the impact of water deficits at the silking stage of maize growth. In contrast, few studies have focused on how the number of maize kernels is affected by water deficits during the vegetative growth period. This study found that water deficits before the silking stage could significantly reduce kernel numbers; the effects became stronger over time (Table 1). The reduction in kernel number was likely to be related to the differentiation and development of reproductive organs under water stress (Fuad-Hassan et al., 2008). Setter and Flannigan (2001) showed that water deficits at an early post-pollination stage could lead to decreases in kernel weight by inhibiting cell division and endoreduplication in the endosperm of maize. Compared with the kernel number, the kernel weight of maize was more significantly affected by the water deficits occurring before silking (NeSmith and Ritchie, 1992a; Moser et al., 2006). The reduced leaf area limited the leaf source activity under water deficits prior to silking during the grain-filling period. This resulted in reduced kernel weight. The water deficit in the vegetative growth period could impact late ovary development; this effect could still be detected during grain-filling (Figure 5). This result has also been confirmed in many other crops (Yang et al., 2009; Hasan et al., 2011; Castillo et al., 2017). Therefore, ovary size is likely a reference indicator for the final kernel weight of maize under abiotic stresses. Hypothetically, ovary sensitivity to water stress is likely associated with the drought tolerance of different maize varieties. This is a topic that should be further examined in future studies.

## Author Contributions

PW and YL designed the project. BZ and YL conducted the field experiment and collected data. YL made the sections and took microphotos and analyzed data. SH and YL drew the tables and figures. SH, HT, and YL drafted the manuscript.

## Conflict of Interest Statement

The authors declare that the research was conducted in the absence of any commercial or financial relationships that could be construed as a potential conflict of interest.
