# Supplementary material for: Timing of Water Deficit Limits Maize Kernel Setting in Association With Changes in the Source-Flow-Sink Relationship
Source: Front Plant Sci. 2018 Oct 22;9:1326. doi: 10.3389/fpls.2018.01326 (PMC6204571; doi:10.3389/fpls.2018.01326)
Supplement: Supplementary file 1 [file Table_1.DOCX]

**Supplementary data**

**Table S1.**

The leaf area at the ear layer, the above ear layer, and the ear and above layer around the tasseling result from water deficit during 2014-2016. CK, well irrigated treatment; V_6-8_, water deficit during the 6-8 leaf stage; V_9-12_, water deficit during the 9-12 leaf stage; V_13-T_, water deficit from the 13-leaf stage to tasseling, R_1-2_, water deficit from the silking stage to blister. *** indicates significance at 0.001.

| Year | Treatments | Leaf area (×10^-2^ m^2^) | | |
| --- | --- | --- | --- | --- |
|  |  | Ear layer | Above ear layer | Ear and above layer |
| 2014 | CK | 23.2 a | 14.9 ab | 38.1 a |
|  | V_6-8_ | 21.2 b | 15.8 a | 37.0 a |
|  | V_9-12_ | 17.1 c | 9.69 c | 26.7 c |
|  | V_13-T_ | 20.8 b | 10.3 c | 31.1 b |
|  | R_1-2_ | 22.2 ab | 13.2 b | 35.4 a |
| 2015 | CK | 24.3 a | 19.0 a | 43.3 a |
|  | V_6-8_ | 21.2 b | 17.9 a | 39.2 b |
|  | V_9-12_ | 19.3 b | 12.7 b | 32.0 c |
|  | V_13-T_ | 22.1 b | 12.6 b | 34.6 c |
|  | R_1-2_ | 24.0 a | 18.7 a | 42.7 ab |
| 2016 | CK | 26.5 a | 12.1 ab | 38.6 ab |
|  | V_6-8_ | 25.3 ab | 11.7 ab | 37.0 bc |
|  | V_9-12_ | 22.9 c | 10.8 bc | 33.8 cd |
|  | V_13-T_ | 24.0 bc | 8.18 c | 32.2 d |
|  | R_1-2_ | 26.9 a | 14.0 a | 40.9 a |
| ANOVA | |  |  |  |
| Year | | *** | *** | *** |
| Treat | | *** | *** | *** |
| Year×Treatment | | ns | ns | ns |

Values with the different letters are significantly different at *P*<0.05. ns indicates no statistically significant relationship.

**Figure captions**

**Fig. S1.** Relative soil water content at three soil layers, 0–20, 20–40 and 40–60 cm during 2014-2016. DAS: days after sowing; CK, well irrigated treatment; V_6-8_, water deficit during the 6-8 leaf stage; V_9-12_, water deficit during the 9-12 leaf stage; V_13-T_, water deficit from the 13-leaf stage to tasseling, R_1-2_, water deficit from the silking stage to blister. Bars: standard deviation of mean value from replications.

**Fig. S2.** Schematic diagram of included leaves at different ear layers. The ear layer include ear leaf, one leaf above it, and one leaf below it; the above layer include all leaves above ear layer; the ear and above layer include leaves at both ear and above layers.

**Fig. S3.** Schematic diagram of logistic curve (black color), its first (red color) and second (blue color) derivation curve, and the definition of characteristic parameters of the grain filling process. The kernel growth logistic function was expressed as $W=a/(1+be^{-ct})$, the first derivation curve of the logistic function was $\frac{dw}{dx}=\frac{abce^{-ct}}{1+be^{-ct}}$, and the second derivation curve of logistic function was $\frac{d^{2}w}{dx^{2}}=\frac{c^{2}abe^{-ct}\left( be^{-ct}-1 \right)}{{(1+be^{-ct})}^{3}}$. The parameter *a* represents the potential kernel weight, b and c are coefficients, which were determined by regression, and t_1_ and t_2_ represent the first and second inflection points of the logistic equation that correspond to the starting and ending time of the active grain filling, respectively.
