# Supplementary figures and images for: Timing of Water Deficit Limits Maize Kernel Setting in Association With Changes in the Source-Flow-Sink Relationship
Source: Front Plant Sci. 2018 Oct 22;9:1326. doi: 10.3389/fpls.2018.01326 (PMC6204571; doi:10.3389/fpls.2018.01326)

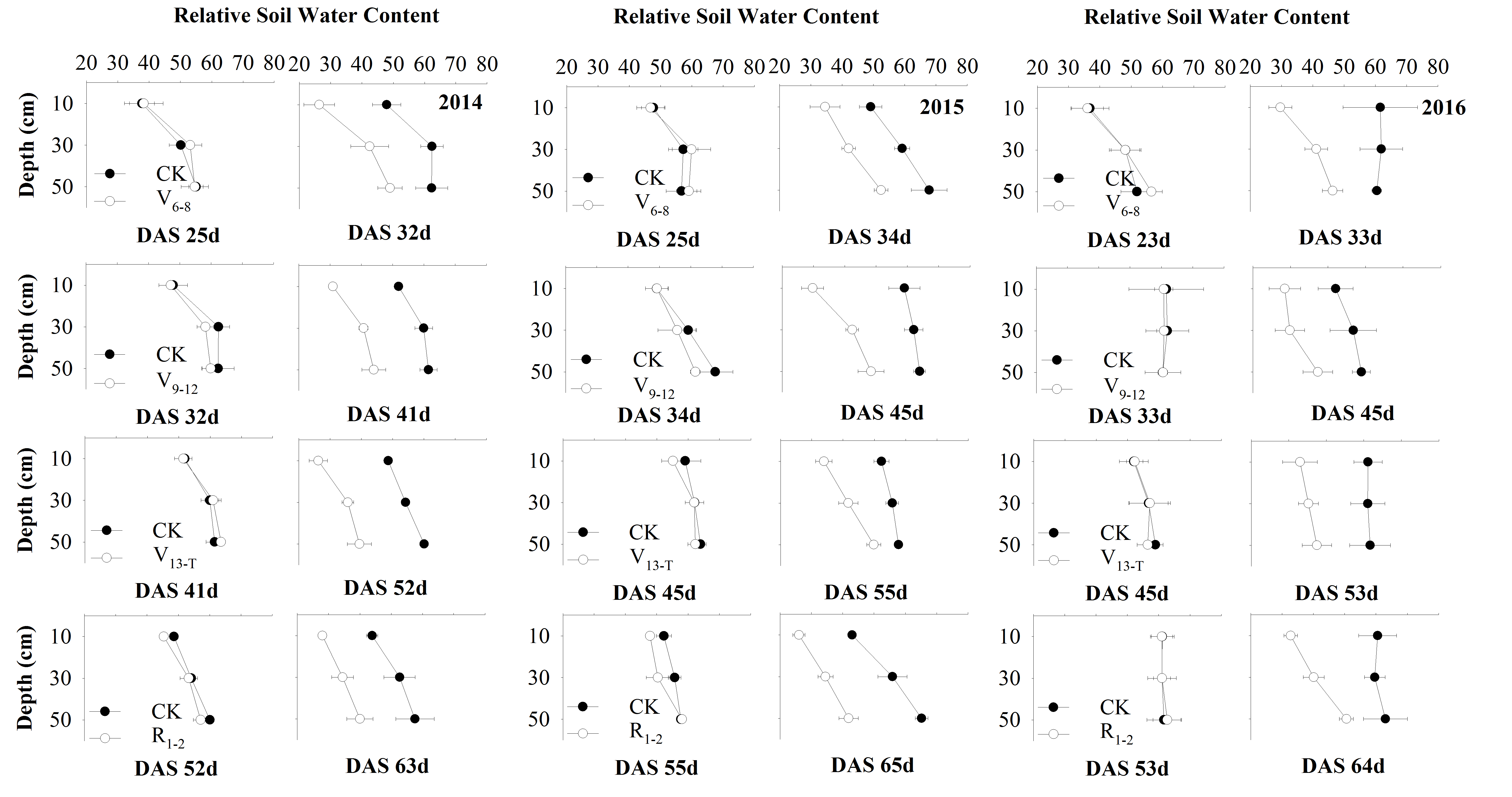

Supplement: Supplementary file 2 [file Image_1.TIF]

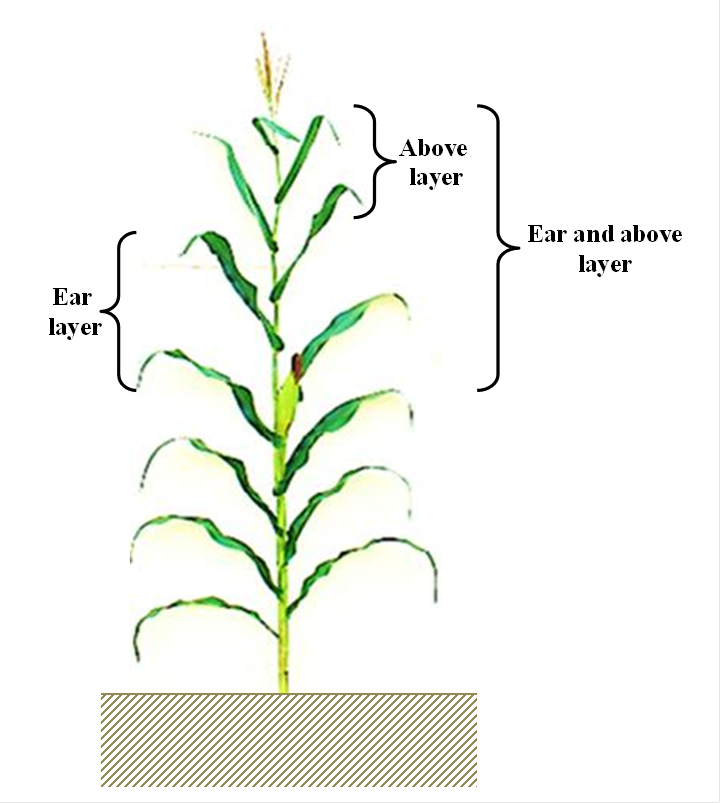

Supplement: Supplementary file 3 [file Image_2.TIF]

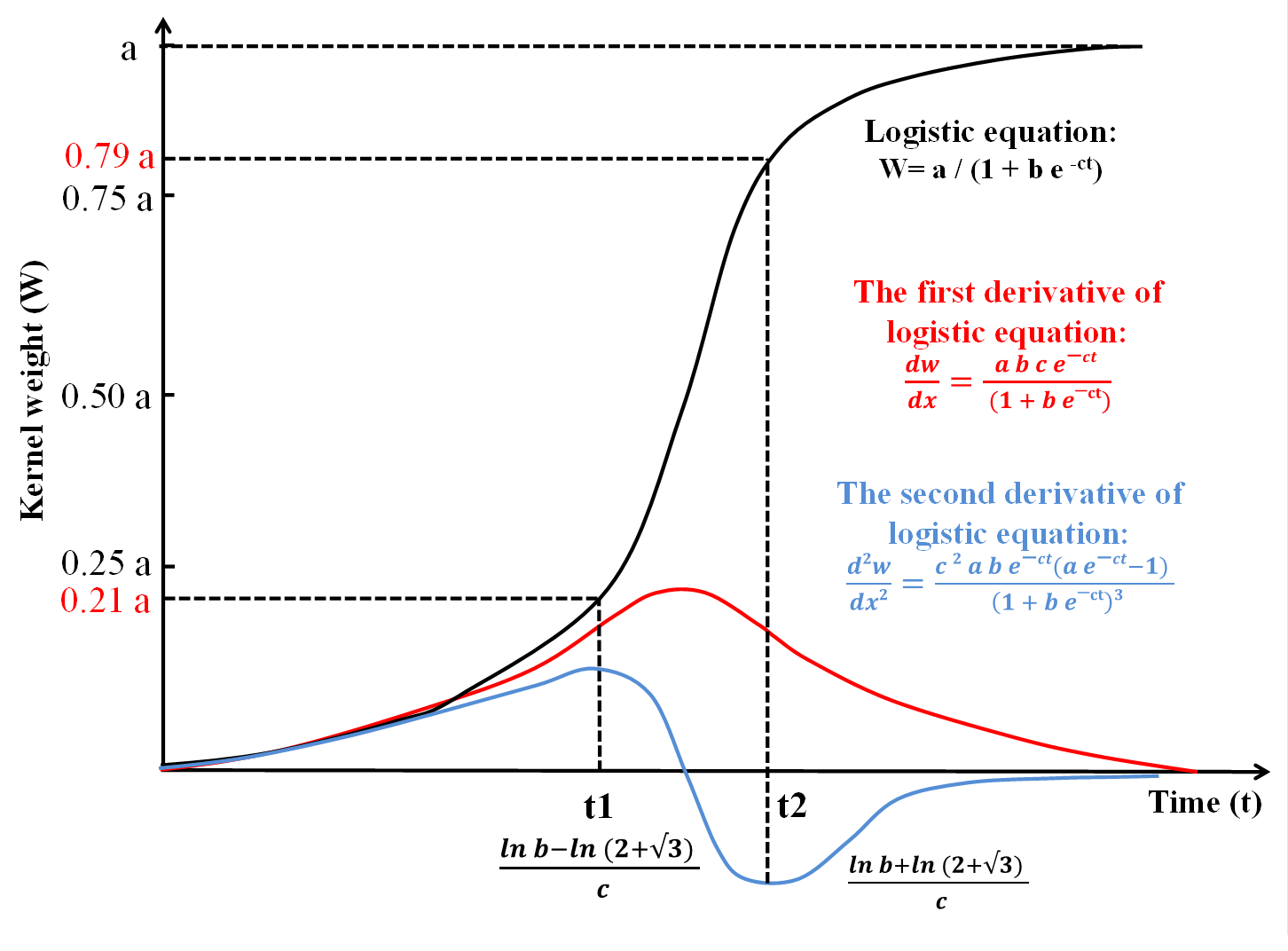

Supplement: Supplementary file 4 [file Image_3.TIF]
